# NITPicker: selecting time points for follow-up experiments

**DOI:** 10.1186/s12859-019-2717-5

**Published:** 2019-04-02

**Authors:** Daphne Ezer, Joseph Keir

**Affiliations:** 1 Department of Statistics, University of Warwick, Coventry, CV4 7AL UK; 2 The Alan Turing Institute, London, NW1 2DB UK; 3 Department of Applied Mathematics and Theoretical Physics, University of Cambridge, Cambridge, CB3 0WA UK

**Keywords:** Time series, Longitudinal, Experimental design, Functional data analysis, RNA-seq, Dynamics

## Abstract

**Background:**

The design of an experiment influences both what a researcher can measure, as well as how much confidence can be placed in the results. As such, it is vitally important that experimental design decisions do not systematically bias research outcomes. At the same time, making optimal design decisions can produce results leading to statistically stronger conclusions. Deciding *where* and *when* to sample are among the most critical aspects of many experimental designs; for example, we might have to choose the time points at which to measure some quantity in a time series experiment. Choosing times which are too far apart could result in missing short bursts of activity. On the other hand, there may be time points which provide very little information regarding the overall behaviour of the quantity in question.

**Results:**

In this study, we develop a tool called NITPicker (Next Iteration Time-point Picker) for selecting optimal time points (or spatial points along a single axis), that eliminates some of the biases caused by human decision-making, while maximising information about the shape of the underlying curves. NITPicker uses ideas from the field of functional data analysis. NITPicker is available on the Comprehensive R Archive Network (CRAN) and code for drawing figures is available on Github (https://github.com/ezer/NITPicker).

**Conclusions:**

NITPicker performs well on diverse real-world datasets that would be relevant for varied biological applications, including designing follow-up experiments for longitudinal gene expression data, weather pattern changes over time, and growth curves.

**Electronic supplementary material:**

The online version of this article (10.1186/s12859-019-2717-5) contains supplementary material, which is available to authorized users.

## Background

**Fig. 1:**
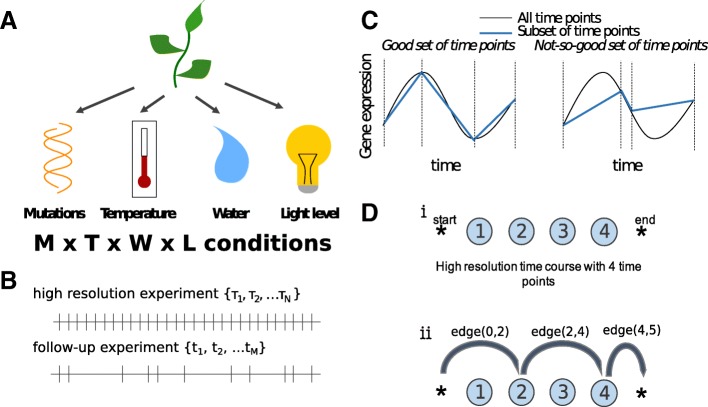
Time point selection is an important part of experimental design. **a** Here is an example of a multi-factor experimental design. **b** Given a set of high resolution time courses, sampled at **τ**_*n*_ we try to find a subset of time points **t**_*m*_ for future follow-up investigations. **c** In this paper, we define good time points as those that enable us to best infer the shape of the function. **d** The time points in a high resolution time course are represented by nodes. In the case shown (i), there are four time points in the high resolution time course. (ii) In the follow-up experiment, time points 2 and 4 are chosen, corresponding to a particular path through the network. The length of this path is the sum of *edge*(0,2), *edge*(2,4) and *edge*(4,5)

In many areas of experimental science, scientists are interested in the behaviour of some system under a wide range of conditions. For instance, a plant biologist might be interested in measuring gene expression in a set of mutant plant varieties under various environmental conditions, such as varying temperature, watering treatments and light intensities, in what is called a factorial (or multi-factor) experimental design (Fig. 1a), but these types of experiments can be very expensive [1].

To complicate matters even more, there are a large number of experiments which do not simply measure some discrete quantity, but instead aim to measure a *function*. Typically, researchers are interested in the behaviour of a quantity *over time* (or, in some cases, space). For example, many genes’ expression levels vary over time in intricate ways, especially since genes are often expressed in bursts. The shape of the burst provides insight into the regulatory mechanisms governing it [2, 3]. Even the degree to which a gene is sensitive to an environmental condition is often time-dependent; for instance, there are a different set of *Arabidopsis thaliana* genes that are sensitive to light at night and during the day [4].

Ideally, a scientist would want to sample at a large number of time points under each experimental condition, but this might not be feasible, especially if the experiments are expensive to run. In such circumstances, the scientist might conduct a small number of high resolution time course experiments, and then use the information gathered to select a subset of time points for further investigation under the entire range of experimental conditions (Fig. 1b). For example, many high resolution time course experiments have recently been published as part of large projects or consortia, including the high resolution time courses of fruit fly [5], roundworm [6], or mammalian lung development [7], but a small lab that is interested in repeating the experiment under slightly different conditions might not be able to afford to use as many time points. Choosing the right subset of time points is clearly important from the point of view of accuracy, but it will also determine what *kinds* of gene expression perturbations can be observed in the follow-up experiments. For example, if a gene were to have a burst of expression in the first experiment, it would be reasonable for the biologist to select the time point corresponding to the peak of the burst for follow-up experiments (Fig. 1c). Then, if the gene shifts the timing of its peak gene expression in the experimental condition, this change would be detected by the experiment, although it would not be possible to relate this to a change in the timing of the peak expression using this measurement alone. On the other hand, if the peak gene expression is the same, but the shape of the distribution changes, the biologist would not be able to detect any change at all (Fig. 1c). Clearly, it would be beneficial to select time points that help us accurately determine the full gene expression profile, while remaining sensitive to the expected types of perturbations.

In this paper, we develop a new statistical tool, called NITPicker, which selects informative time points for follow-up experiments given a set of example curves from a high resolution time course (Fig. 1d). NITPicker uses methods from functional data analysis to find these optimal points, and improves on current approaches for selecting time points for follow-up experiments. The growing field of functional data analysis is focused on developing new statistical techniques to analyse data sampled from continuous curves [8, 9]. In our case, in order to determine the relative importance of each time point for follow-up experiments, we need to know what types of curves we might observe under different experimental conditions. If all possible curves are equally likely to be observed in the new experimental conditions, then any set of time points would be equally sensible to select for the follow-up experiments. In reality, we can expect the observed curves to arise from some non-uniform probability density function of curves, whose parameters we must attempt to infer from the example curves that are available.

Some previous methods for selecting time points for follow-up experiments imagine that all the biological material is collected at each of the original time points and stored, but that the material from each time point is sequenced sequentially based on previous outcomes [10, 11]. However, this is rarely a practical experimental strategy, as it can result in a large amount of wasted time and effort, since biological material is collected at every time point, including those points which are not used in the later analysis. Also, sequencing in parallel can be much quicker and less expensive than sequencing sequentially.

The recent Time Point Selection (TPS) method developed by [12] is a substantial improvement in that it does not depend on this sequential experimental design strategy, and it considers the full shape of the gene expression profile, a strategy also used by NITPicker (Fig. 1d). However, it has three downsides that might limit its use in practice. First, it uses a greedy search strategy for finding time points, which might be prone to finding local optima rather than global optima. In NITPicker, we identify that an optimisation problem described by [12] is in fact the same as a simpler problem in computer science – finding the shortest path through a directed acyclic graph – which can be solved directly by a dynamic programming algorithm (specifically, a modified Viterbi algorithm).

Second, TPS attempts to find the time points which lead to the most accurate fit *for the data in the training set*, so it might not generalise to new gene expression profiles that differ even slightly from the training set. As such, it is useful for follow-up experiments which attempt to repeat the original experiment at a lower resolution. However, in many experimental situations, we are interested in selecting the time points which provide the most information about *how the curve changes* in experimental conditions. For this reason, in NITPicker we use the training set to develop a probability distribution of gene expression curves [13], which allows us to address the slightly different (and more frequently encountered) question of finding the optimal time points for detecting and modelling *perturbations* of the data.

Third, TPS directly uses gene expression profiles from the high resolution time course to select the new time points, a strategy which is potentially vulnerable to experimental noise. On the one hand, we can imagine a scenario in which, for some period of time, the data is very noisy, before later settling down. In this case, TPS is almost certain to select time points in the noisy region (allowing us to more accurately model the noise), despite this providing very little useful information. On the other hand, individual anomalies in the data might cause TPS to select the associated time points. Since NITPicker uses a probability density over gene expression curves [13], this decreases the risk of overfitting the training set, avoiding the latter problem. We also adapt NITPicker for use in the former scenario, by fitting the *inverse coefficient of variation* rather than the data itself.

Ji and Muller [14] developed a similar method to TPS, which has expanded utility since it can find the best time points for performing a linear regression, something that we do not address in this manuscript. Another benefit is that they smooth the functions using functional Principle Component Analysis (fPCA), which we also apply. However, they also suffer from some of the same issues as TPS. Namely, (i) they do not find a probability distribution in the space of functions, but instead fit to the input data directly and (ii) they use either an exhaustive search that is extremely slow or a greedy algorithm that does not guarantee optimality, rather than a dynamic programming algorithm that finds the optimal solution in a reasonable runtime.

In summary, NITPicker does not get trapped in local optima, addresses a wider range of experimental design questions, and is less sensitive to noise in the training set.

## Results

### NITPicker can select time points that describe the shape of a curve

NITPicker is a tool that uses a small number of high resolution time course experiments to select a small set of time points to analyse in follow-up experiments. In order to determine how well NITPicker performs in practice, we apply it to three real-world examples that address the three different experimental design questions corresponding to the *f*_1_, *f*_2_ and *f*_3_ metrics defined the “Methods” section. More specifically, these metrics correspond with the goals of selecting a subset of time points that i) accurately describe the shape of the curve ii) accurately describe the difference between an experimental and control condition and iii) perform goals (i) and (ii) but with preference towards regions of the time series that are less noisy.

**Fig. 2:**
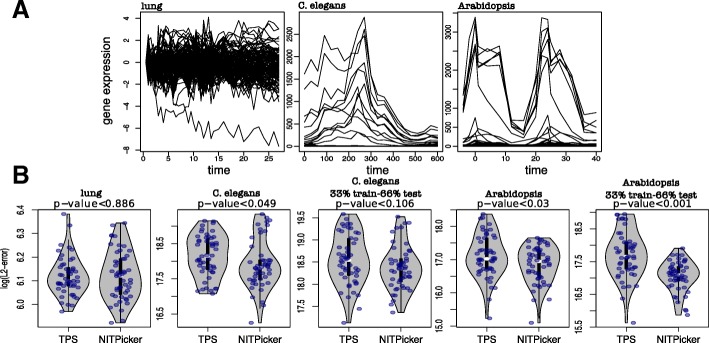
Time point selection for gene expression data. **a** The gene expression curves in the lung, *C. elegans*, and *Arabidopsis* datasets used to test NITPicker. **b** After selecting 8 time points using the training data, the log(L2-error) was calculated on the test data. The training and testing sets were split 50-50, unless specified

First, we test the performance of NITPicker when minimising *f*_1_, which means that we are trying to accurately model the shape of the curves. As this is the same problem proposed by [12], we compared the performance of NITPicker to their TPS method, using three different datasets – the same lung gene expression dataset used in [12], the gene expression of direct targets of the circadian clock gene ELF4 in a longitudinal *Arabidopsis* study across two days at different temperatures [15], and the gene expression profiles of developmental genes in a *C. elegans* developmental time-course [6] (Fig. 2a). In this case, we are presented with an RNA-seq experiment in only one experimental condition, but containing a large number of genes. Within each dataset, the selected subset of genes have similar functional roles, so a reasonable hypothesis appears to be that the corresponding gene expression curves are all drawn from a single probability distribution over curves. To determine if this is an accurate model, we randomly split the dataset into two equal partitions of genes – a training set and a testing set. We then use NITPicker or TPS to select the a subset of eight time points based on the training set, and calculate the L2-error on the genes in the testing set. We repeated this procedure fifty times for each dataset. NITPicker performs equally well as TPS on the less structured lung data, and significantly better than TPS on the more structured *Arabidopsis* and *C. elegans* datasets. This result held even when only a third of the data was used for training and two-thirds was used for testing (Fig. 2b).

### NITPicker can select time points that distinguish between experimental and control conditions

In functional data analysis, two of the standard datasets for testing new algorithms are the Canada weather dataset [8] and the Berkeley growth dataset [16], both of which provide additional examples of real world data with a functional form. Biologists are often interested in how genes and environments interact to produce phenotypes. We have already shown how NITPicker can be applied to gene expression data, but now we show that it can be applicable in understanding environmental data (such as temperature over time) and phenotypic data (growth curves).

**Fig. 3:**
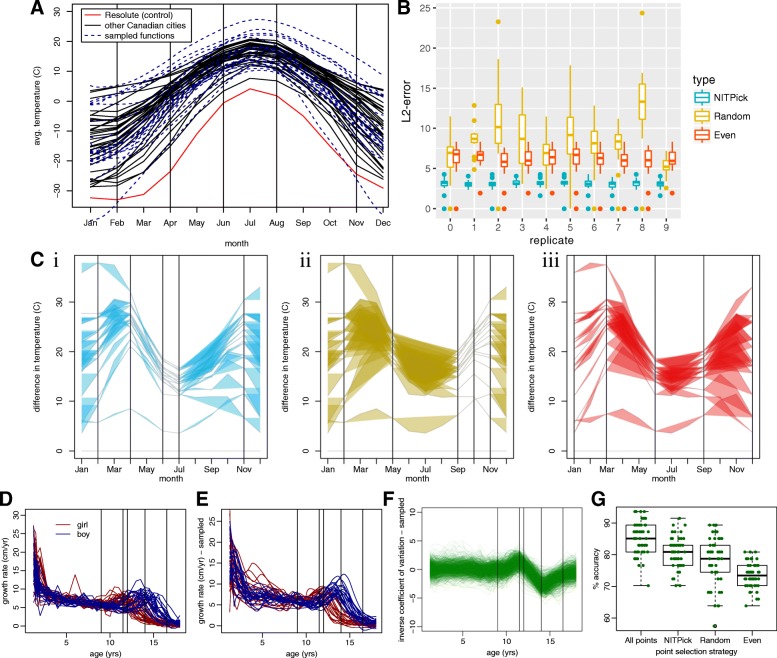
Applying NITPicker. **a** Monthly temperatures from a set of cities in Canada are shown in black. For the purpose of this paper, we consider the ‘control’ condition to be the temperature of Resolute, Canada, which is shown in red. A probability distribution of curves was constructed on the basis of the temperature curves for all cities – curves sampled from this probability distribution are indicated in dashed-blue lines. The vertical lines represent the ‘best time points’ to sample from, according to NITPicker. **b** For each of ten replicates, we selected the time points to sample using half the city curves, and scored the selection of time points on the other half of the city curves. For each city in the test set, we calculated the *L*^2^-error between the curve generated by sampling every month and the curve generated by linear interpolation between the selected subset of time points. **c** Here we present an example of how we evaluate a test set for a selection of time points (vertical bars) selected by NITPicker (i), random (ii), and evenly sampled (iii). The coloured-in area displays the error that arrises from sampling only at the designated time points. **d** The growth rate of boys and girls from the Berkeley growth dataset were used to develop probability distributions of curves for boys and girls, with sampled curves shown in **e**. **f** We were interested in estimating the shape of the inverse coefficient of variation, shown in this figure. The selected time points are shown as vertical bars in D-F. **g** We used half the boy curves and half the girl curves to select time points to sample from, and to train a DD-classifier [18, 19], and then calculated the percent accuracy on the other half of the boy and girl curves. This procedure was repeated 30 times with each method of selecting time points (selecting all the time points, 5 time points with NITPicker, 5 time points randomly, and 5 time points evenly)

The Canada dataset contains the average temperature measured each month across a number of Canadian cities, and we use this to test NITPicker’s performance when minimising *f*_2_ (see “Defining good time points” subsection in the methods section for the definitions of the mathematical quantities referred to here). Suppose that we are interested in the difference in weather between cities in Canada and Resolute, one of its most northerly and coldest cities. In other words, we will be finding the value of *f*_2_, where *g* is the weather of Resolute and *w* is the weather of the other Canadian cities. Suppose (for the sake of illustrating our methods) that we are in a position to sample the weather patterns in some new cities in Canada, but we can only afford to measure the weather in 5 of the 12 months. The raw data is shown in Fig. 3a, showing that we can generate a sensible probability density function for temperature curves in Canada. The best months to sample according to NITPicker are drawn as vertical lines.

To test the accuracy of the approach, we randomly split the cities into two equally sized groups – a training set and a testing set. We use the training set to select a subset of time points using NITPicker, and then we test the strength of these time points on the test set of cities. More specifically, we evaluate the *L*^2^-distance between the curves produced by sampling at all points and those produced by sampling only at the time points selected by NITPicker. We find that NITPicker-selected time points perform better on the testing set than either randomly sampled points or evenly sampled points (Fig. 3b), an example illustrating this is shown in Fig. 3c. This demonstrates that NITPicker can be used successfully to select time points that help distinguish between a control curve and a distribution of curves from experimental conditions.

### NITPicker can avoid time points with noisy values

Growth curves are important in a number of different disciplines in biology, from childhood development [16] to plant sciences [17]. The third dataset represents growth data from a group of boys and girls (Fig. 3d) – despite the unusual shape of the curves, it is possible to develop a reasonable probability distribution of growth curves (Fig. 3e). The largest variance in growth rates is found in the early years; however, from the point of view of distinguishing the two populations, the most informative difference in growth rates between boys and girls is seen during adolescence. Suppose that we want to sample at time points that can help us accurately determine the difference in growth rates between girls and boys. In other words, we don’t mind if the shape of the curve is less accurate in periods of time with lots of variability, but we wish to accurately estimate the shape of the difference between girls and boys in periods of time with less variability within each population – for this we are interested in minimising *f*_3_ (see Fig. 3f, and see “Defining good time points” subsection for the definition of *f*_3_). The point of this exercise is to select time points that help us estimate the shape of the difference between girl and boy curves; however, as by-product of the procedure we might hope that we can select time points that are reasonable at predicting whether an individual growth curve comes from a boy or a girl. Similar to our analysis for the Canada dataset, we split the curves into training and testing sets, but this time we not only select a set of time points using the training set, but we also train a classifier commonly used to classify functional data [18, 19]. Although, as expected, the best classifier used all the time points, NITPicker-selected time points could be used to develop a more accurate classifier than selecting time points either evenly or randomly (Fig. 3g).

## Discussion

In this paper we have presented NITPicker, an algorithm for selecting a subset of time points in time course experiment in a variety of experimental design situations. In contrast to previous strategies [10–12], NITPicker takes full advantage of the *functional* nature of the data to produce a non-parametric probability distribution over curves [13], which is then used to select the optimal time points. This approach minimises the risk of over-fitting the data, while also being better adapted to the situation in which the new time points are being selected for use in experiments which are run under *different conditions* to those used to collect the original data.

The goal of NITPicker is to select points so that, if you interpolate between data sampled at these time points, you form an accurate representation of the underlying curve. This goal is distinct from the closely related, but fundamentally different goal of finding a small set of points that allow the researcher to reconstruct the rest of the curve, which is sometimes referred to as *trajectory reconstruction* (Additional file 1: Figure S1a). For instance, it is possible that a time point’s value might be highly correlated to a much later time point’s value, so that in theory only one of these time points must be sampled, and the value at the second time point can be inferred. In this case, a method such as the one proposed by [14] might suggest sampling at a smaller number of time points; however, a downside is that the scientist would not be able to directly observe the curve, so they would need to trust that the previously observed correlations between time points will continue to hold under new experimental conditions. If the goal of the scientist is to sample at a few points, and then reconstruct the underlying curve using a predetermined method (such a spline fitting), then NITPicker is the appropriate tool.

Similarly, NITPicker does not find the time points that are the best time points for distinguishing between control groups and experimental groups, because the goal is to accurately characterise the profile of the temporal differences between the experimental and control group over time. Many biologists are not interested in diagnosing the type of exposure, but are instead interested in learning how the experimental groups differ from one another. However, we show that as a side effect, the time points that are selected can distinguish between experimental groups better than random or evenly selected time points (Fig. 3). If the goal of the researcher is to be able to distinguish between experimental and control groups, a binary classifier would be a more appropriate tool.

NITPicker is especially designed to find good time points for new experiments, so if there are lots of temporal shifts in the curves it will find time points that will be useful in the case of similar types of temporal shifts (Additional file 2: Figure S2b). This might be especially useful for researchers who study systems where the same features are expected to appear in all samples over the time series, but the timing of those features might vary.

The output of NITPicker depends on which curves are included in the analysis. To ensure that the selected time points are most reflective of biologically interesting behaviours, the biologist can choose to only include curves that are less noisy or that are most relevant for the biological question at hand. For example, a biologist might focus on genes that are regulated by a certain transcription factor or that have a specific gene ontology of interest. To filter noisy curves, it might be a good idea to use a tool like edge [20] to identify curves that smoothly change their level over the time course. Finally, a biologist may have some strategy to quantify the importance of genes, and these weights can be provided to NITPicker. NITPicker can take as few as three high resolution time series as input, and this creates reasonable probability distributions of simple curves (See Additional file 1: Figure S1). However, it is important to always visualise the probability distributions of curves using the *getPerturbation* function in the NITPicker package, to ensure that these seem to accurately reflect the observed data. The more high resolution curves are available under different experimental conditions, the more likely it will be that the optimal time points are selected for future experiments.

NITPicker uses a dynamic programming algorithm to select the optimal time points, which provides an efficient method that is guaranteed to find the optimal solution (unlike the greedy algorithm used in TPS [12]). One of the downsides of using this dynamic programming algorithm is that it only outputs the predicted optimal set of time points, but does not provide an easy way of evaluating the confidence in this prediction. We tested NITPicker on a variety of simulated and real-life datasets, and demonstrated the flexibility of this tool by addressing different experimental design questions in each case.

## Conclusion

NITPicker provides a useful tool for selecting informative time points that avoids the pitfalls of ad hoc human decisions. Specifically, it can select the optimal time points to address a variety of different experimental goals, including accurately predicting the shape of a curve, accurately predicting the perturbations to some base-line curve, or selecting time points which are most informative (i.e. avoiding regions of random noise). By choosing time points using NITPicker, researchers are likely to obtain results with more statistical power, while at the same time avoiding bias.

## Methods

### Defining good time points

In order to compare the strengths and weaknesses of each heuristic, we need to clearly define of what constitutes a *good* time point to select. One possible strategy would be to try to select a set of time points that are best able to distinguish the shape of the curves. For this we select a criteria very similar to that presented by [12] and [14] in that we want to minimise the *L*^2^-distance between the curve generated by sampling at all the time points, and the curve generated by sampling only at a subset of time points. However, the other methods minimise this distance over all curves in the training set – instead, we use the training set to generate a probability density over the space of curves, and then minimise the expected distance. Some of the advantages of this approach have already been mentioned in the introduction.

First, we will describe the functions that the NITPicker algorithm minimises, so that the overall goal of NITPicker is clear from the beginning. We will start by describing the input functions (*w*) in terms of a set of parameters (*μ*). However, there are many ways of parameterising a function – for instance, any function can be represented as a linear combination of a Fourier, B-spline, or polynomial basis, and the parameters that specify the function would be the relevant coefficients (such as *a*_0_, *a*_1_, and *a*_2_ in *y*(*t*)=*a*_0_+*a*_1_*x*(*t*)+*a*_2_*x*^2^(*t*) if the function was a quadratic function). At this stage, we do not describe how the functions are parameterised, but our choice is described in detail in “Defining a non-parametric probability density function of curves” section. In brief, we parameterise the curves by the functional principle components of the x-axis and y-axis deformations from the mean curve.

Suppose that we have an initial high resolution time course, with data sampled at times ***τ***={*τ*_1_,*τ*_2_,...*τ*_*N*_}. We must select a subset of these time points, which we will call ***t***={*t*_1_,*t*_2_,...*t*_*M*_}, so as to minimise the expected error:

1 $$ f_{1}(\boldsymbol{t}):= \int ||w(t;\boldsymbol{\tau}, \mu)-w(t;\boldsymbol{t}, \mu) ||^{2}_{L^{2}(t,[\tau_{1},\tau_{N}])}P(\mu) \mathrm{d} \mu  $$

where *w*(*t*;***t***,*μ*) is a gene expression function evaluated at the time *t*, parameterised by a set of parameters *μ* and interpolated between time points in the set ***t*** (either through a linear interpolation or spline), and *P*(*μ*)d*μ* is a probability measure on the space of parameters (i.e. *P*(*μ*) is the probability density associated with the set of parameters *μ*). We use the standard notation for *L*^2^ norms, that is, given a function *w*(*t*;*μ*) of time *t* and some parameters *μ* we define

$$||w(t;\mu)||_{L^{2}(t, [\tau_{1}, \tau_{2}])} := \left(\int_{\tau_{1}}^{\tau_{2}} \left(w(t;\mu)\right)^{2} \mathrm{d} t \right)^{\frac{1}{2}} $$

In many cases we are not necessarily interested in the shape of the curve, but rather the difference between the control and an experimental condition. Let *g*(***t***,*ν*) be the gene expression curve in the control condition, parameterised by *ν* and sampled at time points *t*. Then we want to minimize the expected error in the difference:

2 $$ \begin{aligned} f_{2}(\boldsymbol{t}) &:= \iint|| \left(g(t;\boldsymbol{t}, \nu)-w(t;\boldsymbol{t}, \mu)\right) -(g(t;\boldsymbol{\tau}, \nu) \\ &\quad-w(t;\boldsymbol{\tau}, \mu)) ||^{2}_{L_{2}(t,[\tau_{1},\tau_{N}])} P_{1}(\mu)P_{2}(\nu)\mathrm{d}\mu \mathrm{d}\nu \end{aligned}  $$

If there is only one ‘control’ curve (for instance, if the scientists have not included replicates), then there would only be one possible value for the parameters *ν* and the equation is simplified to:

3 $$ \begin{aligned} & f_{2}(\boldsymbol{t}) := \int|| \left(g(t;\boldsymbol{t})-w(t;\boldsymbol{t}, \mu)\right) \\ &\phantom{\sum_{m=1}^{M}\int_{\nu} \int_{\mu}||} -\left(g(t;\boldsymbol{\tau})-w(t;\boldsymbol{\tau}, \mu) \right) ||^{2}_{L_{2}(t,[\tau_{1},\tau_{N}])} P(\mu) \mathrm{d}\mu \end{aligned}  $$

Note that *f*_1_ is a special case of *f*_2_ where *g*(*t*;***t***)=0.

If there are periods of time with different amounts of random variability, then we might wish to sample less frequently in areas that have lots of variability – we might accept having less accuracy in predicting the shape of the curves in noisy regions if we can accurately model their shapes in regions with less noise. To accomplish this, we should attempt to find the shape of the curve representing the difference between the control and experimental conditions *normalised by the variance*. In other words, we minimise the expected error in the *inverse of the coefficient of variation*. We first define *z*:

4 $$ \begin{aligned} &z(t;\boldsymbol{t}, \boldsymbol{\tau}, \nu, \mu) := \left(g(t;\boldsymbol{t}, \nu)-w(t;\boldsymbol{t}, \mu)\right) \\ &\phantom{z(t;\boldsymbol{t}, \boldsymbol{\tau}, \nu, \mu) :=} - \left(g(t; \boldsymbol{\tau}, \nu)-w(t;\boldsymbol{\tau}, \mu) \right) \end{aligned}  $$

and then:

5 $$ \begin{aligned} &f_{3}(\boldsymbol{t}):= \\ &\iint \bigg|\bigg| \frac{z(t;\boldsymbol{t}, \boldsymbol{\tau}, \nu, \mu)}{ \sqrt{\text{Var} (z(t;\boldsymbol{t}, \boldsymbol{\tau}, \nu, \mu))}} \bigg|\bigg|^{2}_{L_{2}(t, [\tau_{1}, \tau_{N}])} P_{1}(\mu)P_{2}(\nu)\mathrm{d}\mu \mathrm{d}\nu \end{aligned}  $$

where the variance is itself a function of *t*: it is the variance of the function *z*(*t*;***τ***,***t***,*ν*,*μ*) with respect to the probability measure *P*_1_(*μ*)d*μP*_2_(*ν*)d*ν*.

There are many criteria that might be used to determine the “optimal” set of time points to select, but for the purpose of this manuscript we focus on these three criteria, as they are intuitive, relatively simple, and – as we shall see later – the best solution can be computed exactly with a dynamic programming algorithm. Note also that our algorithm can easily be adapted to deal with many other criteria. For example, if it is important to accurately measure both the curve *and its firsts* derivatives, then we can simply replace the *L*^2^ norms in the above expressions with the norms associated with the Sobolev spaces *H*^*s*^. Additionally, if the scientist can quantify the *importance* of each gene, they can add weights, for instance by multiplying each *x*_*i*_(*t*) in *f*_1_ by its corresponding weight.

### Defining a non-parametric probability density function of curves

In order to effectively find the subset of time points that minimise *f*_1_, *f*_2_, or *f*_3_, we need a method to derive, from a set of example curves, the probability of observing particular curves in future experiments.

First, suppose that we have chosen some way of parameterising curves using a set of parameters *μ*. Let *g*_*a*_(*t*) be the curve produced in the *a*-th high resolution time course experiment, and let $\mu _{g_{a}}$ be the associated parameters. Given the whole set of such parameters associated with all the high resolution time courses, we want to generate a probability density function *P*(*μ*) on the parameter space.

In some cases, a scientist might have a model in mind that describes the functions – in this case they can directly fit the parameters. However, in most cases we’ve encountered scientists do not have such a model, nor can one be justified on theoretical grounds, so we need a *non-parametric* way of defining a probability distribution of functions given a set of examples. In the discipline of functional data analysis, techniques have been developed to define these probability distributions – see [13] – a process that involves first aligning the functions (“registration”) and then parameterising the horizontal and vertical shifts (using functional Principle Component Analysis). Both the horizontal and vertical shifts are parameterised, so curves that are sampled from the probability density function of curves will have similar x-axis and y-axis deformations as the original curves. For completeness, we will summarise their protocol below (Additional file 3: Figure S3).

First, we take a set of known functions (our set of high resolution functions, *g*_*a*_(*t*)) and *align* them. In order to effectively align the gene expression curves in a shape preserving way, we define a distance between curves in terms of the square root slope function (SRSF):

$$q(t)=\text{sign}(\dot{f}(t))\sqrt{|\dot{f}(t)|} $$

Note that, given *q*(*t*) and the initial value *f*(0), we can recover the corresponding function *f*(*t*) via $f(t)=f(0)+\int _{0}^{t}{q(s)|q(s)|}ds$.

Now, we define the *y*-distance between functions *h*_1_ and *h*_2_ as

6 $$  D_{y}(h_{1}, h_{2}):=\inf_{\gamma \in \Gamma}||q_{1}(t)-(q_{2} \circ \gamma)(t) \sqrt{\dot{\gamma}(t)}||_{L^{2}(t, [\tau_{1}, \tau_{n}])}  $$

where *γ* is a function defining the amount of *x*-axis warp, and *Γ*⊂*L*^2^ is the set of *warping functions*. *γ*∈*Γ* must have some special properties: (i) *γ*(*t*)∈[0,1] (ii) *γ* is monotonically increasing (i.e its slope is positive) (iii) $||\gamma (t)||_{L^{2}(t)}=1\phantom {\dot {i}\!}$. Because of this last point, the warping functions must lie on the *unit sphere in* *L*^2^, which can be thought of as an infinite-dimensional sphere.

Given a function *g*_(mean)_ (see [13] for the appropriate *g*_(mean)_), a dynamic programming algorithm is used to find a vector of functions ***γ***=(*γ*_*a*_(*t*)), where *γ*_*a*_ corresponds to the warping function found when computing *D*_*y*_(*g*_(mean)_,*g*_*a*_) (see Eq. 6). It also provides us with a set of aligned functions *f*_*a*_ and a corresponding set of aligned *q* functions *q*_*a*_. SRSF is a continuous alignment algorithm that ensures that the aligned curves are differentiable, so it does not lead to alignment artefacts observed when applying feature registration or discrete dynamic time warping algorithms.

We would like use the set of warping functions *γ*_*a*_ to define a probability density function on (some subset of) *Γ*. However, *Γ* is not a linear space: given two warping function *γ*_1_ and *γ*_2_, their sum *γ*_1_+*γ*_2_ cannot be interpreted as a warping function, since it will not lie on the unit sphere in *L*^2^. Hence, we cannot immediately apply functional Principal Component Analysis. Instead, we linearise the space *Γ* by first finding the centroid of the points *γ*_*a*_ on the surface of the sphere (the “Karcher mean”, *γ*_(mean)_). Note that this is itself a function of *t*. Next, we use the *exponential map* at *γ*_(mean)_, which provides us with a map from the tangent space at *γ*_(mean)_ (which *is* a linear space) to the sphere itself. More precisely, given a tangent vector to the sphere, we find the point on the sphere reached by exponentiating this tangent vector, using the Lie-group structure (under composition) of the unit sphere in *L*^2^. In this way, we can associate a vector in the tangent space at *γ*_(mean)_ to each of the warping functions *γ*_*a*_. This linearisation step should not affect small perturbations, but may exaggerate the differences between pairs of outliers– any two curves that differ substantially from the other curves will be considered farther apart from each other than in reality.

Now, to decrease the dimensionality of the space (and to decrease the number of free parameters in the model), we can perform a functional Principle Component Analysis (fPCA) of this linearised space, then fit an independent normal distribution along each principle axis. This gives us a probability density on the tangent space. Finally, we can use the exponential map again to map this probability density function directly onto the sphere.

An fPCA can also be performed on the y-axis deformations. This time, the functions defining the *y*-axis deformations are simply functions in *L*^2^, which is already a linear space, so we don’t need to perform the linearisation step.

fPCA is a method that finds an eigenbasis of functions (principle components), and orders them by the amount of variance that they can explain. Each function can then be expressed as a linear combination of the top principle component functions, plus an error term. Neglecting the error term has the effect of smoothing the resulting curves. Therefore, this step also makes the method more resilient to experimental noise. One of the reasons we treat x-axis and y-axis deformations separately is that fPCA only considers variance in terms of y-axis deformations, not x-axis deformations. Ji and Muller [14] also uses fPCA, but without separating the x- and y-axis deformations first. Also, [14] use it for smoothing, rather than for parameterising a probability density of functions.

In summary, this strategy results in *μ* being defined as the space of fPCA coordinates, associated with both *x*- and *y*-deformations. *P*(*μ*) is then given by the multivariate normal distribution with a diagonalised covariance matrix.

To estimate the integrals involved in the definitions of *f*_1_, *f*_2_ or *f*_3_, we take *J* samples from the probability distributions defined above. For example, we estimate *f*(***t***) as

$$f_{\text{est}}(\boldsymbol{t})= \frac{1}{J} \sum_{j=1}^{J} || w(t; \boldsymbol{\tau}, \mu_{j}) - w(t; \boldsymbol{t}, \mu_{j}) ||^{2}_{L^{2}(t, [\tau_{1},\tau_{N}])} $$

where *μ*_*j*_ is the set of parameters corresponding to the *j*-th sample drawn from *P*(*μ*). By default we set J to 1000. Note that since we are minimising *f*, the factor of $\frac {1}{J}$ can be dropped.

The benefit of this approach is that it allows the user define very complicated functions for *w*(*g*,*μ*) and *P*(*μ*), and still be able to apply NITPicker. The downside of this approach is its random nature, which means that we don’t know the error between the actual value of the integral and this estimate, although we can be confident that *f*_(est)_ is close to *f* if *J* is sufficiently large.

### NITPicker algorithm

A dynamic programming algorithm can be employed to find the set of *m* time points ***t*** that minimise *f*(***t***) (where *f*=*f*_1_, *f*_2_ or *f*_3_). In essence, the problem is identical to finding the path that minimises the distance in a directed acyclic graph that contains exactly *m* edges, which can be calculated with a modified Viterbi algorithm (Fig. 1d). Consider a graph with *N*+2 ordered nodes – a ‘start’ node, *N* nodes that represent each time point in the high resolution time course, and an ‘end’ node. For ease of notation, we index the start node with 0 and the end node with *N*+1. Each node is connected by edges that point to all the nodes that are ahead of it, and we set the value of the edge joining node *i* to node *k* to be:

7 $$ \textit{edge}(i, k)=\sum_{j=1}^{J}||[w(t; \boldsymbol{\tau}, \mu_{j}) - w(t; \{\tau_{i}, \tau_{k}\}, \mu_{j})]||^{2}_{L^{2}(t, [\tau_{i},\tau_{k}])}  $$

In other words, the value of the edge joining node *i* to node *k* is the *L*^2^-error caused by selecting times *τ*_*i*_ and *τ*_*k*_ and none of the times in between.

Now we need to find the shortest path with *K* edges that goes from the start node to the end node. An *N* by *N* by *K* table can be assembled where each element is:

8 $$ \Theta(i, j, k)=\min_{\ell = 0,1,\ldots,i} \Big(\Theta(\ell, j-1, k-1)+\textit{edge}(i, j) \Big)  $$

and *Θ*(0,*j*,0)=0, *Θ*(*i*>0,*j*,0)=*∞*. In other words, the value of *Θ*(*i*,*j*,*k*) is the minimum error when going from *τ*_0_ to *τ*_*i*_ and then immediately to *τ*_*j*_, using exactly *k* edges.

The value of *f*_est_(***t***) is *Θ*(*N*+1,*N*+1,*K*). As we construct the table *Θ*, we also save another matrix *Θ*_*min*_, with entries given by *Θ*_*min*_(*j*,*k*)= min*iΘ*(*i*,*j*,*k*). The value of *Θ*_*min*_(*j*,*k*) is then the minimum *L*^2^-error when going from *τ*_0_ to *τ*_*j*_ using *k* steps. We also construct a similar *N* by *K* matrix *Θ*_*trace*_(*j*,*k*), with entries given by the value of *i* which minimises *Θ*(*i*,*j*,*k*). This can then be used to find the time points: we set *t*_*k*_=*Θ*_*trace*_(*N*+1,*k*), and then *t*_*k*−1_=*Θ*_*trace*_(*t*_*k*_,*k*−1).

Note that the value of *edge*(*i*,*j*) actually depends on the previous *R* time points if a spline of degree *R* is used. However, the index of the previous *R* best nodes given edge (*i*,*j*) can be easily computed from the traceback matrix, although this makes NITPicker run much more slowly. Furthermore, using a spline of degree greater than one can produce edge-effects, especially in the beginning of the sequence as we use the deBoor algorithm to calculate the spline [21]. This problem can be reduced by running the dynamic programming algorithm twice – once forward and once backwards.

## Additional files

Additional file 1 **Figure S1**: Examples of probability densities of functions with three example curves. NITPicker can be run with as few as three high resolution time courses. In order to see if it performs reasonably under these circumstances, we randomly chose triplets of skewed Gaussian curves with varied means (right), skews (middle) and standard deviations (left) and these are shown in red. Sampled curves (100) from the probability density of functions are shown in grey. These seem like reasonable predictions given the input data. (PDF 422 kb)

Additional file 2 **Figure S2**: Why a biologist might want to use the time point selection criteria used by NITPicker. The smooth black and red curves show the original high resolution time course data. The horizontal dotted lines indicate the time points that might be selected under the indicated method. (A) NITPicker selects points that describe the shape of the curve, rather than points that can be used to reconstruct the curve. It might be possible to infer the shape of the curve with fewer points than suggested by NITPicker (top), but you would not have direct observations as to the shape of the curve (unlike NITPicker–bottom), so this relies on greater trust that the model will continue to hold under new experimental conditions. (B) Other methods find the best time points for the observed data, so they might overfit (top). In this case, NITPicker would observe that there is a lot of variability in the peak in the early time course and would pick more evenly spaced time points in this region for follow up experiments (bottom). (PDF 18 kb)

Additional file 3 **Figure S3**: Flowchart of algorithm for generating a probability densities of functions. This is the exact same procedure used by [13], but is included here for completeness. First, the curves are aligned in order to dissect and quantify the x-axis and y-axis shifts in the curves. Then, these x-axis and y-axis shifts are parameterised by their functional Principle Components, and this is used to generate curves that have similar x-axis and y-axis shifts to the original curves. (PDF 11 kb)

## Abbreviations

CRAN: Comprehensive R archive network
fPCA: Functional principle component analysis
NITPicker: Next iteration time-point picker
SRSF: Square root slope function
TPS: Time point selection
