# Supplementary figures and images for: NITPicker: selecting time points for follow-up experiments
Source: BMC Bioinformatics. 2019 Apr 2;20:166. doi: 10.1186/s12859-019-2717-5 (PMC6444531; doi:10.1186/s12859-019-2717-5)

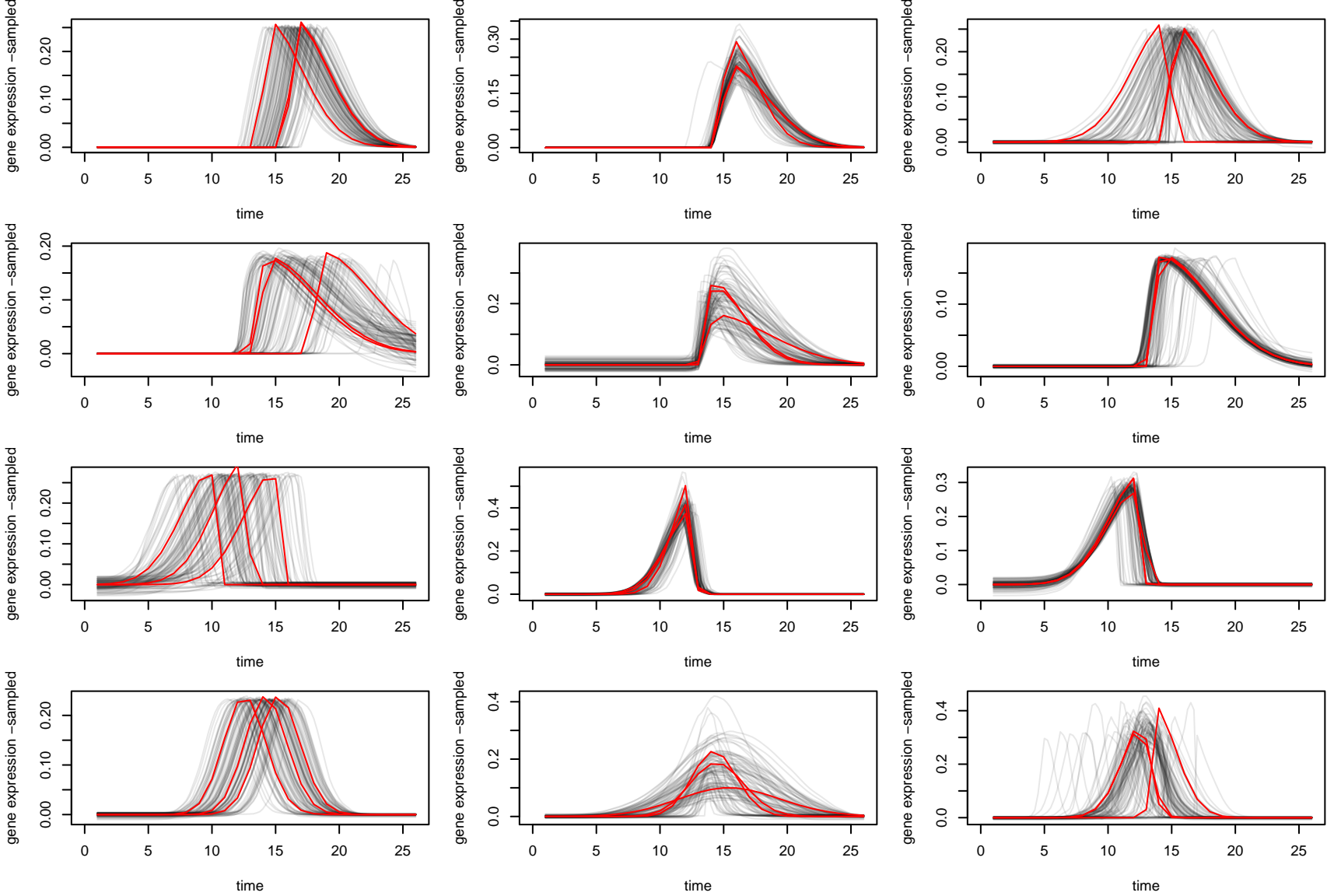

Supplement: Supplementary file 1 — Figure S1: Examples of probability densities of functions with three example curves. NITPicker can be run with as few as three high resolution time courses. In order to see if it performs reasonably under these circumstances, we randomly chose triplets of skewed Gaussian curves with varied means (right), skews (middle) and standard deviations (left) and these are shown in red. Sampled curves (100) from the probability density of functions are shown in grey. These seem like reasonable predictions given the input data. (PDF 422 kb) [file 12859_2019_2717_MOESM1_ESM.pdf]

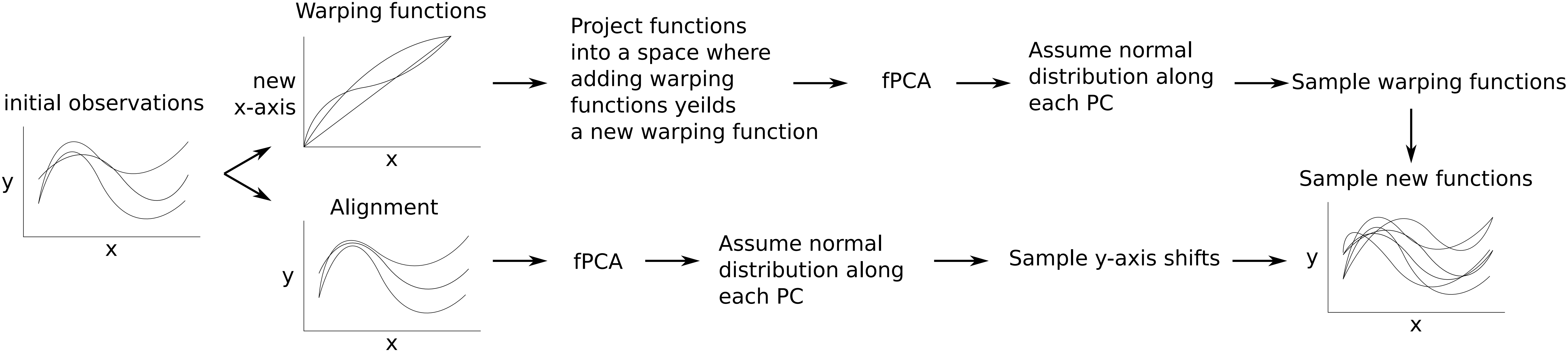

Supplement: Supplementary file 3 — Figure S3: Flowchart of algorithm for generating a probability densities of functions. This is the exact same procedure used by [13], but is included here for completeness. First, the curves are aligned in order to dissect and quantify the x-axis and y-axis shifts in the curves. Then, these x-axis and y-axis shifts are parameterised by their functional Principle Components, and this is used to generate curves that have similar x-axis and y-axis shifts to the original curves. (PDF 11 kb) [file 12859_2019_2717_MOESM3_ESM.pdf]
