# Supplementary material for: NITPicker: selecting time points for follow-up experiments
Source: BMC Bioinformatics. 2019 Apr 2;20:166. doi: 10.1186/s12859-019-2717-5 (PMC6444531; doi:10.1186/s12859-019-2717-5)
Supplement: Supplementary file 2 — Figure S2: Why a biologist might want to use the time point selection criteria used by NITPicker. The smooth black and red curves show the original high resolution time course data. The horizontal dotted lines indicate the time points that might be selected under the indicated method. (A) NITPicker selects points that describe the shape of the curve, rather than points that can be used to reconstruct the curve. It might be possible to infer the shape of the curve with fewer points than suggested by NITPicker (top), but you would not have direct observations as to the shape of the curve (unlike NITPicker–bottom), so this relies on greater trust that the model will continue to hold under new experimental conditions. (B) Other methods find the best time points for the observed data, so they might overfit (top). In this case, NITPicker would observe that there is a lot of variability in the peak in the early time course and would pick more evenly spaced time points in this region for follow up experiments (bottom). (PDF 18 kb) [file 12859_2019_2717_MOESM2_ESM.pdf]

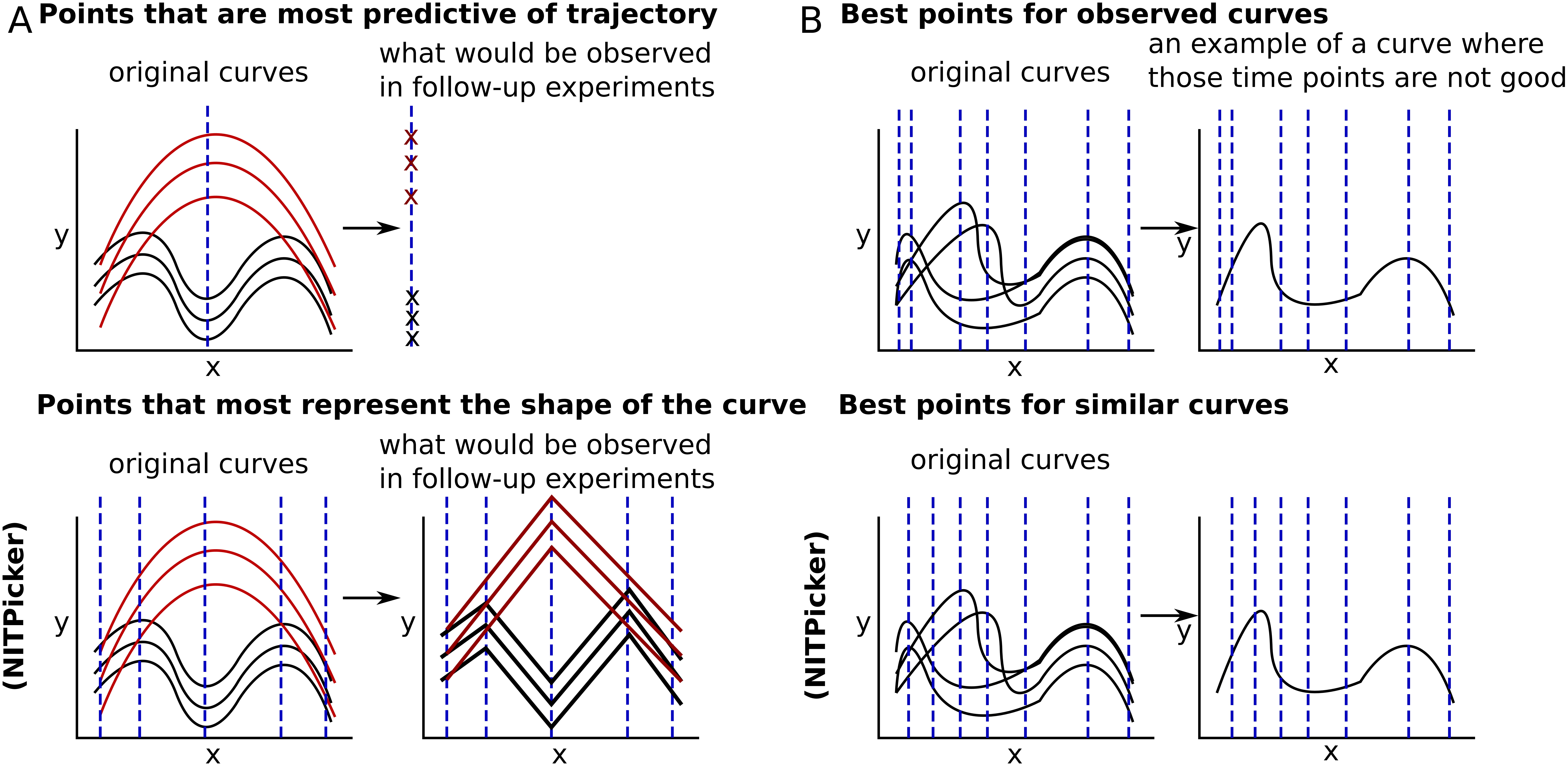
A
Points that are most predictive of trajectory
original curves
what would be observed
in follow-up experiments
y
x
Points that most represent the shape of the curve
(NITPicker)
original curves
what would be observed
in follow-up experiments
y
x
B
Best points for observed curves
original curves
an example of a curve where
those time points are not good
y
x
Best points for similar curves
(NITPicker)
original curves
y
x
